# Author Correction: Single human B cell-derived monoclonal anti-*Candida* antibodies enhance phagocytosis and protect against disseminated candidiasis

**DOI:** 10.1038/s41467-019-08392-x

**Published:** 2019-01-18

**Authors:** F. M. Rudkin, I. Raziunaite, H. Workman, S. Essono, R. Belmonte, D. M. MacCallum, E. M. Johnson, L. Silva, A. S. Palma, T. Feizi, A. Jensen, L. P. Erwig, N. A. R. Gow

**Affiliations:** 1 Medical Research Council Centre for Medical Mycology at the University of Aberdeen, Aberdeen, AB25 2ZD UK; 2 Global Biotherapeutic Technologies, Pfizer Inc., Cambridge Kendall Square, Cambridge, 02139 MA USA; 3 National Infection Service, PHE South West Laboratory, Science Quarter, Southmead Hospital, Bristol, BS10 5NB UK; 4 Glycosciences Laboratory, Department of Medicine, Imperial College London, Du Cane Road, W12 0NN UK; 5 UCIBIO-REQUIMTE, Department of Chemistry, Faculty of Science and Technology, NOVA University of Lisbon, Lisbon, 1099-085 Portugal; 6 Present Address: Division of Infection and Immunity, The Roslin Institute and Royal (Dick) School of Veterinary Studies, University of Edinburgh, Edinburgh, EH25 9RG UK; 7 Present Address: HiFiBiO, 325 Vassar Street, Cambridge, MA 02139 USA; 8 Present Address: MSD Animal Health Innovation AS, Thormøhlensgate 55, N-5006 Bergen, Norway; 9 Present Address: H. Lundbeck, Ottiliavej 9, 2500 Valby, Denmark; 10 Present Address: Galvani Bioelectronics, 980 Great West Road, Brentford, TW8 9GS UK; 11 Present Address: School of Biosciences, University of Exeter, Geoffrey Pope Building, Exeter, EX4 4QD UK

Correction to: *Nature Communications*; 10.1038/s41467-018-07738-1; published online 11 December 2018

The original version of this Article contained errors in the author affiliations.

The present address of the author Ingrida Raziunaite, ‘Division of Infection and Immunity, The Roslin Institute and Royal (Dick) School of Veterinary Studies, University of Edinburgh, Edinburgh EH25 9RG, UK’ was incorrectly assigned as a full affiliation.

The present address of the author Sosthene Essono, ‘HiFiBiO, 325 Vassar Street, Cambridge, MA 02139, USA’ was incorrectly assigned as a full affiliation.

The present address of the author Rodrigo Belmonte, ‘MSD Animal Health Innovation AS, Thormøhlensgate 55, 5006 Bergen, Norway’ was incorrectly assigned as a full affiliation.

The present address of the author Allan Jensen, ‘H. Lundbeck, Ottiliavej 9, 2500 Valby, Denmark’ was incorrectly assigned as a full affiliation.

The present address of the author Lars P. Erwig, ‘Galvani Bioelectronics, 980 Great West Road, Brentford TW8 9GS, UK’ was incorrectly assigned as a full affiliation.

The present address of the author Neil A.R. Gow, ‘School of Biosciences, University of Exeter, Geoffrey Pope Building, Exeter EX4 4QD, UK’ was incorrectly assigned as a full affiliation.

In addition, the affiliation of Ingrida Raziunaite with Medical Research Council Centre for Medical Mycology at the University of Aberdeen, Aberdeen Fungal Group, Institute of Medical Sciences, Foresterhill, Aberdeen AB25 2ZD, UK was inadvertently omitted.

The affiliation of Sosthene Essono with Global Biotherapeutic Technologies, Pfizer Inc., 610 Main Street, Kendall Square, Cambridge, MA 02139, USA was inadvertently omitted.

The affiliation of Rodrigo Belmonte with Medical Research Council Centre for Medical Mycology at the University of Aberdeen, Aberdeen Fungal Group, Institute of Medical Sciences, Foresterhill, Aberdeen AB25 2ZD, UK was inadvertently omitted.

The affiliation of Allan Jensen with Global Biotherapeutic Technologies, Pfizer Inc., 610 Main Street, Kendall Square, Cambridge, MA 02139, USA was inadvertently omitted.

The affiliation of Lars P. Erwig with Medical Research Council Centre for Medical Mycology at the University of Aberdeen, Aberdeen Fungal Group, Institute of Medical Sciences, Foresterhill, Aberdeen AB25 2ZD, UK was inadvertently omitted.

The affiliation of Neil A.R. Gow with Medical Research Council Centre for Medical Mycology at the University of Aberdeen, Aberdeen Fungal Group, Institute of Medical Sciences, Foresterhill, Aberdeen AB25 2ZD, UK was also inadvertently omitted.

These errors have been corrected in both the PDF and HTML versions of the Article.

