# Supplementary material for: Single human B cell-derived monoclonal anti-Candida antibodies enhance phagocytosis and protect against disseminated candidiasis
Source: Nat Commun. 2018 Dec 11;9:5288. doi: 10.1038/s41467-018-07738-1 (PMC6290022; doi:10.1038/s41467-018-07738-1)
Supplement: Supplementary file 1 — Supplementary Information [file 41467_2018_7738_MOESM1_ESM.pdf]

Supplementary information

Single human B cell-derived monoclonal anti-*Candida* antibodies enhance phagocytosis and protect against disseminated candidiasis

Rudkin et al.

Supplementary Table 1 – Clinical isolates and strains used in this study.

**Strain name**	**Genotype**	**Reference**
CAI4+CIp10 (NGY152)	*ura3Δ::λimm434/ura3Δ::λimm434 RPS1/rps1::URA3*	(1)
hyr1Δ	*hyr1Δ::hisG/hyr1Δ:hisG-URA-3-hisG*	(2)
hyr1Δ+HYR1	*hyr1::hisG/hyr1::hisG/RPS1/rps1::HYR1*	(this work)
tup1Δ	*tup1Δ::hisG/tup1Δ::hisG-URA3-hisG*	(3)
C. albicans SC5314	Clinical isolate	(4)
C. glabrata SCS71182B	Clinical isolate	(5)
C. tropicalis AM2005/0546	Clinical isolate	Clinical isolate from Aberdeen Royal Infirmary
C. lusitaniae SCS211362H	Clinical isolate	(5)
C. krusei SCS71987M	Clinical isolate	(5)
C. parapsilosis ATCC22019	Clinical isolate	(6)
C. dubliniensis CD36	Clinical isolate	(7)
A. fumigatus V05-27	Clinical isolate	(8)
C. auris CBS 10913T	Clinical isolate	(9)
C. haemulonii CBS 5149T	Clinical isolate	(10)
C. neoformans KN99α	H99 mating type α	(11)
C. gattii R265	Clinical isolate	(12)
P. carinii M167-6	Isolated from rat lung tissue	Theodore J. Kottom & Andrew Limper (Mayo Clinic College of Medicine, Rochester)
S. cerevisiae NCPF8313	Clinical isolate	Mycology Ref Lab, Bristol

Supplementary Table 2 – Primers for Reverse Transcription-Polymerase Chain Reaction

Primer	Sequence 5'-3'	Heavy/kappa/lambda
L-VH1 (forward)	ACAGGTGCCCACTCCCAGGTGCAG	Heavy
L-VH3 (forward)	AAGGTGTCCAGTGTGARGTGCAG	Heavy
L-VH4/6 (forward)	CCCAGATGGGTCCTGTCCCAGGTGCA G	Heavy
L-VH5 (forward)	CAAGGAGTCTGTTCCGAGGTGCAG	Heavy
CgCH1 (reverse)	GGAAGGTGTGCACGCCGCTGGTC	Heavy
IgG internal (reverse)	GTTCGGGGAAGTAGTCCTTGAC	Heavy
L-Vk1/2 (forward)	ATGAGGSTCCCYGCTCAGCTGCTGG	Kappa
L-Vk3 (forward)	CTCTTCCTCCTGCTACTCTGGCTCCCA G	Kappa
L-Vk4 (forward)	ATTTCTCTGTTGCTCTGGATCTCTG	Kappa
Ckappa3UTRCt_Rev (reverse)	TTC TCC TCC AAC ATT AGC ATA AT	Kappa
Ckappa3UTRNt_Rev (reverse)	TGG AAC TGA GGA GCA GGT G	Kappa
RT Kc Rev (reverse)	ACA CTC TCC CCT GTT GAA GCT C	Kappa
L-VL1 (forward)	GGTCCTGGGCCCAGTCTGTGCTG	Lambda
L-VL2 (forward)	GGTCCTGGGCCCAGTCTGCCCTG	Lambda
L-VL3 (forward)	GCTCTGTGACCTCCTATGAGCTG	Lambda
L-VL4/5 (forward)	GGTCTCTCTCSCAGCYTGTGCTG	Lambda
L-VL6 (forward)	GTTCTTGGGCCAATTTTATGCTG	Lambda
L-VL7 (forward)	GGTCCAATTCYCAGGCTGTGGTG	Lambda
L-VL8 (forward)	GAGTGGATTCTCAGACTGTGGTG	Lambda
Clambda3UTRCt_Rev (reverse)	TTT ATT GAG GGT TTA TTG AGT GC	Lambda
Clambda3UTRNt_Rev (reverse)	AGC TCT AGT CTC CCG TGG TG	Lambda
RTLC12367Rev (reverse)	TGA ACA TTC TGT AGG GGC CAC TGT	Lambda

Supplementary Table 3 – Nested PCR primers for VH

Primer	Sequence 5'-3'	Forward/reverse
BssHIIVHFW-1	ACA GCT ACA GGC GCG CAC TCC CAG ATG CAG CTG GTG CAA TCT GG	Heavy forward
BssHIIVHFW-2	ACA GCT ACA GGC GCG CAC TCC CAG ATG CAG CTG GTG CAG TCT GG	Heavy forward
BssHIIVHFW-3	ACA GCT ACA GGC GCG CAC TCC CAG GTG ACC TTG AAG GAG TCT GG	Heavy forward
BssHIIVHFW-4	ACA GCT ACA GGC GCG CAC TCC GAG GTG CAG CTG GTG CAG TCT G	Heavy forward
BssHIIVHFW-5	ACA GCT ACA GGC GCG CAC TCC SAG GTS AMC TTR ARG CAG TCT G	Heavy forward
BssHIIVHFW-6	ACA GCT ACA GGC GCG CAC TCC GAR GTG CAG CTG GTG SAG TCY G	Heavy forward
BssHIIVHFW-7	ACA GCT ACA GGC GCG CAC TCC CAG GTA CAG CTS SAG CAG TCA GG	Heavy forward
BssHIIVHFW-8	ACA GCT ACA GGC GCG CAC TCC CAG GTG CAG CTG GTG CAA TCT GG	Heavy forward
BssHIIVHFW-9	ACA GCT ACA GGC GCG CAC TCC CAG GTG CAG CTG GTG CAG TCT GG	Heavy forward
BssHIIVHFW-10	ACA GCT ACA GGC GCG CAC TCC CAG GTC CAG CTG GTA CAG TCT GG	Heavy forward
BssHIIVHFW-11	ACA GCT ACA GGC GCG CAC TCC CAG GTC CAG CTG GTG CAG TCT GG	Heavy forward
BssHIIVHFW-12	ACA GCT ACA GGC GCG CAC TCC GAG GTC CAG CTG GTA CAG TCT GG	Heavy forward
BssHIIVHFW-13	ACA GCT ACA GGC GCG CAC TCC GAG GTC CAG CTG GTG CAG TCT GG	Heavy forward
BssHIIVHFW-14	ACA GCT ACA GGC GCG CAC TCC CAG ATC ACC TTG AAG GAG TCT GG	Heavy forward
BssHIIVHFW-15	ACA GCT ACA GGC GCG CAC TCC CAG GTC ACC TTG AAG GAG TCT GG	Heavy forward
BssHIIVHFW-16	ACA GCT ACA GGC GCG CAC TCC GAA GTG CAG CTG GTG GAG TCT GG	Heavy forward
BssHIIVHFW-17	ACA GCT ACA GGC GCG CAC TCC GAG GTG CAG CTG GTG GAG TCT GG	Heavy forward
BssHIIVHFW-18	ACA GCT ACA GGC GCG CAC TCC CAG GTG CAG CTG GTG GAG TCT GG	Heavy forward
BssHIIVHFW-19	ACA GCT ACA GGC GCG CAC TCC GAG GTG CAG CTG GTG GAG ACT GG	Heavy forward
BssHIIVHFW-20	ACA GCT ACA GGC GCG CAC TCC GAG GTG CAG CTG GTG GAG TCC GG	Heavy forward
BssHIIVHFW-21	ACA GCT ACA GGC GCG CAC TCC GAG GTG CAG CTG GTG GAG TCT CG	Heavy forward
BssHIIVHFW-22	ACA GCT ACA GGC GCG CAC TCC GAG GTG CAG CTG TTG GAG TCT GG	Heavy forward
BssHIIVHFW-23	ACA GCT ACA GGC GCG CAC TCC CAG CTG CAG CTG CAG GAG TCG GG	Heavy forward

BssHIIVHFW-24	ACA GCT ACA GGC GCG CAC TCC CAG CTG CAG CTG CAG GAG TCC GG	Heavy forward
BssHIIVHFW-25	ACA GCT ACA GGC GCG CAC TCC CAG GTG CAG CTG CAG GAG TCG GG	Heavy forward
BssHIIVHFW-26	ACA GCT ACA GGC GCG CAC TCC CAG GTG CAG CTA CAG CAG TGG GG	Heavy forward
BssHIIVHFW-27	ACA GCT ACA GGC GCG CAC TCC CAG GTA CAG CTG CAG CAG TCA GG	Heavy forward
VHtraIgG1_Rev1	GAC CGA TGG GCC CTT GGT CGA GGC TGA GGA GAC GGT GAC	Heavy reverse
VHtraIgG1_Rev2	GAC CGA TGG GCC CTT GGT CGA GGC TGA GGA GAC GCT GAC	Heavy reverse
VHtraIgG1_Rev3	GAC CGA TGG GCC CTT GGT CGA GGC TGA GGA GAC GGA GAC	Heavy reverse

Supplementary Table 4 – Kappa primers for nested PCR

Primer	Sequence 5'-3'	Forward/reverse
BssHIIVKFW-1	ACA GCT ACA GGC GCG CAC TCG GAC ATC CAG ATG ACC CAG TCT CC	Kappa forward
BssHIIVKFW-2	ACA GCT ACA GGC GCG CAC TCG GAC ATC CAG TTG ACC CAG TCT CC	Kappa forward
BssHIIVKFW-3	ACA GCT ACA GGC GCG CAC TCG GCC ATC CAG ATG ACC CAG TCT CC	Kappa forward
BssHIIVKFW-4	ACA GCT ACA GGC GCG CAC TCG GCC ATC AGG ATG ACC CAG TCT CC	Kappa forward
BssHIIVKFW-5	ACA GCT ACA GGC GCG CAC TCG GTC ATC TGG ATG ACC CAG TCT CC	Kappa forward
BssHIIVKFW-6	ACA GCT ACA GGC GCG CAC TCG GCC ATC CAG TTG ACC CAG TCT CC	Kappa forward
BssHIIVKFW-7	ACA GCT ACA GGC GCG CAC TCG AAC ATC CAG ATG ACC CAG TCT CC	Kappa forward
BssHIIVKFW-8	ACA GCT ACA GGC GCG CAC TCG GAA ATT GTA ATG ACA CAG TCT CC	Kappa forward
BssHIIVKFW-9	ACA GCT ACA GGC GCG CAC TCG GAA ATT GTG TTG ACG CAG TCT CC	Kappa forward
BssHIIVKFW-10	ACA GCT ACA GGC GCG CAC TCG GAA ATA GTG ATG ACG CAG TCT CC	Kappa forward
BssHIIVKFW-11	ACA GCT ACA GGC GCG CAC TCG GAT AYT GTG ATG ACC CAG ACT CC	Kappa forward
BssHIIVKFW-12	ACA GCT ACA GGC GCG CAC TCG GAT GTT GTG ATG ACT CAG TCT CC	Kappa forward
BssHIIVKFW-13	ACA GCT ACA GGC GCG CAC TCG GAT ATT GTG MTG ACT CAG TCT CC	Kappa forward

BssHIIVKFW-14	ACA GCT ACA GGC GCG CAC TCG GAC ATC GTG ATG ACC CAG TCT CC	Kappa forward
BssHIIVKFW-15	ACA GCT ACA GGC GCG CAC TCG GAA ACG ACA CTC ACG CAG TCT CC	Kappa forward
BssHIIVKFW-16	ACA GCT ACA GGC GCG CAC TCG GAA ATT GTG CTG ACT CAG TCT CC	Kappa forward
BssHIIVKFW-17	ACA GCT ACA GGC GCG CAC TCG GAW RTT GTG CTG ACW CAG TCT CC	Kappa forward
BssHIIVKFW-18	ACA GCT ACA GGC GCG CAC TCG GAC ATT GTG CTG ACC CAG TCT CC	Kappa forward
PTT5_HUK-INF-REV	CCA GAG GTC GAG GTC GGG GGA TCC CTA ACA CTC TCC CCT GTT GAA GCT CTT TG	Kappa reverse

Supplementary Table 5 – Lambda primers for nested PCR

Primer	Sequence 5’-3’	Forward/reverse
BssHIIVLFW-1	ACA GCT ACA GGC GCG CAC TCG CAG TCT GTC CTG ACG CAG CCG CC	Lambda forward
BssHIIVLFW-2	ACA GCT ACA GGC GCG CAC TCG CAG TCT GTC GTG ACG CAG CCG CC	Lambda forward
BssHIIVLFW-3	ACA GCT ACA GGC GCG CAC TCG CAG TCT GTC TTG ACG CAG CCG CC	Lambda forward
BssHIIVLFW-4	ACA GCT ACA GGC GCG CAC TCG TCC TAT GWG CTG ACT CAG CC	Lambda forward
BssHIIVLFW-5	ACA GCT ACA GGC GCG CAC TCG CAG TCT GTG CTG ACG CAG CCG CC	Lambda forward
BssHIIVLFW-6	ACA GCT ACA GGC GCG CAC TCG CAG TCT GTG GTG ACG CAG CCG CC	Lambda forward
BssHIIVLFW-7	ACA GCT ACA GGC GCG CAC TCG CAG TCT GTG TTG ACG CAG CCG CC	Lambda forward
BssHIIVLFW-8	ACA GCT ACA GGC GCG CAC TCG CAG TCT GTG CTG ACT CAG CCA CC	Lambda forward
BssHIIVLFW-9	ACA GCT ACA GGC GCG CAC TCG CAG TCT GCC CTG ACT CAG CCT	Lambda forward
BssHIIVLFW-10	ACA GCT ACA GGC GCG CAC TCG AGC TAT GAG CTG ACT CAG CCA CC	Lambda forward
BssHIIVLFW-11	ACA GCT ACA GGC GCG CAC TCG AGC TAT GAG CTG ACT CAG CCA CT	Lambda forward
BssHIIVLFW-12	ACA GCT ACA GGC GCG CAC TCG AGC TAT GAG CTG ACA CAG CCA CC	Lambda forward
BssHIIVLFW-13	ACA GCT ACA GGC GCG CAC TCG AGT TCT GAG CTG ACT CAG GAC CC	Lambda forward
BssHIIVLFW-14	ACA GCT ACA GGC GCG CAC TCG AGC TAT GTG CTG ACT CAG CCA CC	Lambda forward

BssHIIVLFW-15	ACA GCT ACA GGC GCG CAC TCG AGC TAT GAG CTG ACA CAG CTA CC	Lambda forward
BssHIIVLFW-16	ACA GCT ACA GGC GCG CAC TCG AGC TAT GAG CTG ATG CAG CCA CC	Lambda forward
BssHIIVLFW-17	ACA GCT ACA GGC GCG CAC TCG AGC TAT GAG CTG ACA CAG CCA TC	Lambda forward
BssHIIVLFW-18	ACA GCT ACA GGC GCG CAC TCG CTG CCT GTG CTG ACT CAG CCC CC	Lambda forward
BssHIIVLFW-19	ACA GCT ACA GGC GCG CAC TCG CAG CCT GTG CTG ACT CAA TCA TC	Lambda forward
BssHIIVLFW-20	ACA GCT ACA GGC GCG CAC TCG CAG CTT GTG CTG ACT CAA TCG CC	Lambda forward
BssHIIVLFW-21	ACA GCT ACA GGC GCG CAC TCG CAG CCT GTG CTG ACT CAG CCA CC	Lambda forward
BssHIIVLFW-22	ACA GCT ACA GGC GCG CAC TCG CAG GCT GTG CTG ACT CAG CCG GC	Lambda forward
BssHIIVLFW-23	ACA GCT ACA GGC GCG CAC TCG CAG CCT GTG CTG ACT CAG CCA TC	Lambda forward
BssHIIVLFW-24	ACA GCT ACA GGC GCG CAC TCG AAT TTT ATG CTG ACT CAG CCC CA	Lambda forward
BssHIIVLFW-25	ACA GCT ACA GGC GCG CAC TCG CAG ACT GTG GTG ACT CAG GAG CC	Lambda forward
BssHIIVLFW-26	ACA GCT ACA GGC GCG CAC TCG CAG GCT GTG GTG ACK CAG GAG CC	Lambda forward
BssHIIVLFW-27	ACA GCT ACA GGC GCG CAC TCG CAG ACT GTG GTG ACC CAG GAG CC	Lambda forward
BssHIIVLFW-28	ACA GCT ACA GGC GCG CAC TCG CAG CCT GTG CTG ACT CAG CCA CC	Lambda forward
BssHIIVLFW-29	ACA GCT ACA GGC GCG CAC TCG CAG GCA GGG CTG ACT CAG CCA CC	Lambda forward
BssHIIVLFW-30	ACA GCT ACA GGC GCG CAC TCG CAG CTT GTG CTG ACT CAG YC	Lambda forward
BssHIIVLFW-31	ACA GCT ACA GGC GCG CAC TCG CAG CTC GTG CTG ACT CAG YC	Lambda forward
PTT5_HUL7-INF-REV	CCA GAG GTC GAG GTC GGG GGA TCC CTC AAG AGC ATT CTG CAG GGG CCA CTG TTT G	Lambda reverse
PTT5_HUL1-3-INF-REV	CCA GAG GTC GAG GTC GGG GGA TCC CTC ATG AAC ATT CTG TAG GGG CCA CTG	Lambda reverse

Supplementary Table 6 – Purified recombinant human IgG1 mAbs generated using the single B cell technology.

Antibody	Yield (mg)	Target
AB-120	12	Hyr1 protein
AB-121	28.5	Hyr1 protein
AB-122	67.9	Hyr1 protein
AB-123	67.3	Hyr1 protein
AB-124	38.9	Hyr1 protein
AB-118	7.5	*C. albicans* ‘whole cell’
AB-119	13.5	*C. albicans* ‘whole cell’
AB-126	60.9	*C. albicans* 'whole cell’
AB-127	24.5	*C. albicans* 'whole cell’
AB-129	2.3	*C. albicans* 'whole cell’
AB-131	24.1	*C. albicans* 'whole cell’
AB-132	9.3	*C. albicans* 'whole cell’
AB-133	19	*C. albicans* 'whole cell’
AB-134	7.7	*C. albicans* 'whole cell’
AB-135	16.5	*C. albicans* 'whole cell’
AB-139	12.2	*C. albicans* 'whole cell’
AB-140	19.5	*C. albicans* 'whole cell’

Supplementary Table 7 – Fungal, Bacterial and Plant Polysaccharide Array. Saccharide probes included in the Fungal, Bacterial and Plant polysaccharide array and the fluorescence binding intensities elicited with all the mAbs investigated and Dectin-1 used as a reference in the analysis.

			Fluorescence binding intensities[c]								
**ID**	**Probe[a]**	**Predominant oligosaccharide sequence where known[b]**	**AB118**	**AB119**	**AB121**	**AB126**	**AB127**	**AB131**	**AB135**	**AB140**	**Dectin-1**
1	Dextran *L. mesenteroides*	α1,6-Glc	-	-	-	-	-	366 (149)	-	-	-
2	Pullulan *P. pullulans*	Mixed α1,4/α1,6-Glc	-	13 (86)	-	-	-	4 (24)	-	-	-
3	Curdlan[d] *Agrobacterium* sp.	β1,3-Glc	-	-	-	-	-	-	-	731 (914)	39,104 (800)
4	NSG *S. cerevisiae*		-	-	-	-	-	-	-	-	9,369 (114)
5	PGG *S.cerevisiae*	Linear β1,3-Glc backbone with occasional monoglucosyl β1,6-Glc branches	42 (11)	-	-	-	3 (25)	-	-	-	47,943 (2,874)
6	Lentinan *L. edodes*		-	-	-	-	-	-	-	103 (165)	65,153 (2)
7	Grifolan *G. frondosa*	β1,3-Glc backbone with highly ramified oligomeric β1,6-Glc branches	-	-	-	-	-	-	-	-	4,971 (68)
8	Barley β-glucan	Mixed β1,3/β1,4-Glc	-	-	-	-	-	24 (12)	-	-	-
9	Oat β-glucan	Mixed β1,3/β1,4-Glc	24 (57)	-	44 (56)	26 (21)	-	-	-	-	-
10	Lichenan	Mixed β1,3/β1,4-Glc	-	-	1,337 (1,402)	-	-	-	-	-	51 (2)
11	Pustulan *U. papullosa*	β1,6-Glc	-	-	289 (29)	-	-	-	-	-	21 (3)
12	Mannan *S. cerevisiae*	α1,6-Man backbone with oligomeric α1,2-, α1,3-Man branches (13)	-	-	-	-	-	716 (50)	-	-	-
13	*N*-Mannoprotein *C.albicans*	α1,6-Man backbone with oligomeric α1,2-, α1,3-, and β-1,2-Man branches (13-15)	39,449 (842)	41,714 (437)	-	24,686 (606)	20,663 (215)	22,319 (375)	30,286 (100)	43,194 (157)	913 (33)
14	Mannoprotein *A. fumigatus*	Mannose-rich (16)	-	-	-	-	-	-	-	-	-
15	Lipomannan *M. tuberculosis*	Linear α1,6-Man backbone with monomannosyl α1,2-Man branches (17)	-	-	-	-	-	-	-	-	-
16	Lipoarabinomannan *M. tuberculosis*	Linear α1,6-Man backbone with monomannosyl α1,2-Man branches and α1,5-Ara polymer branched at certain	-	-	-	-	-	-	-	-	-

		positions with α1-3,1-5-Ara residues, which in turn are terminated by β1,2-Ara and capped by α1,2-Man units (17)									
17	Lipoarabinomannan M. smegmatis	Linear α1,6-Man backbone with monomannosyl α1,2-Man branches and α1,5-Ara polymer branched at certain positions with α1-3,1-5-Ara residues, which in turn are terminated by β1,2-Ara and capped by phospho inositol (17)	-	-	-	-	-	-	-	-	-
18	Native *O*-glycoprotein *M. tuberculosis*	α1,2-Man (18)	-	-	-	-	-	-	-	-	-
19	Glucurono-XyloMannan[e] *T. fuciformis*	α1,3-Man with Xyl, GlcA and Fuc branches	-	-	9 (7)	-	-	-	-	-	-
20	GN6-AO[f]	GlcNAcβ-4GlcNAcβ -4GlcNAcβ -4GlcNAcβ -4GlcNAcβ -4GlcNAc-AO	-	-	-	-	-	-	-	44 (134)	3 (5)

[a] Unless otherwise indicated the saccharide probes are polysaccharides: NSG, Neutral soluble β-glucan; PGG, Poly-(1,6)-D-glucopyranosyl-(1,3)- D-glucopyranose

[b] Glc, Glucose; Man, Mannose; Gal, Galactose; Ara, Arabinose; Xyl, xylose; GlcA, Glucuronic acid; Fuc, fucose; GlcNAc, *N*-acetylglucosamine.

[c] These are means of fluorescence intensities of duplicate spots printed at the high level of probe arrayed (0.1ng/spot); '–', less than 1; The numbers in brackets are the errors (half of the difference of signal intensities of duplicate spots for each saccharide/glycan probe).

[d,e] Curdlan polysaccharide was solubilized in 50mM NaOH and Glucurono-XyloMannan in 150 mM NaCl, prior printing.

[f] GN6-AO, neoglycolipid (NGL) probe prepared from reducing hexasaccharide of chitin (β1,4-linked *N*-acetylglucosamine, GlcNAc) by oxime ligation with an aminooxy (AO) functionalized DHPE (19).

Supplementary Table 8 – *N*-glycan Array Set 3. Oligosaccharide probes included in the *N*-glycan microarray and the fluorescence binding intensities elicited with mAb PGT 128 used as a reference in the analysis.

			Fluorescence binding intensities[b]
ID	Probe[a]	Oligosaccharide sequence	PGT 128
1	Man3(α3,α6)	Manα-6Man-DH<br>| Manα-3	49<br>(11)
2	Man5(α3,α6)	Manα-3<br>| Manα-6Manα-6Man-DH<br>| Manα-3	63<br>(63)
3	Man1GN1	Manß-4GlcNAc-DH	56<br>(63)
4	Man2GN1	Manα-3Manß-4GlcNAc-DH	49<br>(10)
5	Man2aGN2	Manα-6Manß-4GlcNAcß-4GlcNAc-DH	-
6	Man3GN2	Manα-6<br>| Manß-4GlcNAcß-4GlcNAc-DH<br>| Manα-3	25<br>(19)
7	Man3XylGN2	Manα-6<br>| Xylß-2Manß-4GlcNAcß-4GlcNAc-DH<br>| Manα-3	73<br>(105)
8	Man3FGN2	Manα-6 Fucα-6<br>| | Manß-4GlcNAcß-4GlcNAc-DH<br>| Manα-3	71<br>(10)
9	Man3FXylGN2	Manα-6<br>| Xylß-2Manα-4GlcNAcß-4GlcNAc-DH<br>| | Manα-3 Fucα-3	-
10	Man4aGN2	Manα-3Manα-6<br>| Manß-4GlcNAcß-4GlcNAc-DH<br>| Manα-3	46<br>(30)
11	Man4bGN2	Manα-6<br>| Manα-3Manα-6<br>| Manß-4GlcNAcß-4GlcNAc-DH	-
12	Man5GN2	Manα-6<br>| Manα-3Manα-6<br>| Manß-4GlcNAcß-4GlcNAc-DH<br>| Manα-3	27<br>(21)

<table>
<tr><td>13</td><td>Man6GN2</td><td>Manα-6
|
Manα-3Manα-6
|
Manß-4GlcNAcß-4GlcNAc-DH
|
Manα-2Manα-3</td><td>46
(22)</td></tr>
<tr><td>14</td><td>Man7(D1)GN2</td><td>Manα-6
|
Manα-3Manα-6
|
Manß-4GlcNAcß-4GlcNAc-DH
|
Manα-2Manα-2Manα-3</td><td>87
(22)</td></tr>
<tr><td>15</td><td>Man7(D3)GN2</td><td>Manα-2Manα-6
|
Manα-3Manα-6
|
Manß-4GlcNAcß-4GlcNAc-DH
|
Manα-2Manα-3</td><td>993
(4)</td></tr>
<tr><td>16</td><td>Man8(D1D3)GN2</td><td>Manα-2Manα-6
|
Manα-3Manα-6
|
Manß-4GlcNAcß-4GlcNAc-DH
|
Manα-2Manα-2Manα-3</td><td>5,186
(124)</td></tr>
<tr><td>17</td><td>Man9GN2</td><td>Manα-2Manα-6
|
Manα-2Manα-3Manα-6
|
Manß-4GlcNAcß-4GlcNAc-DH
|
Manα-2Manα-2Manα-3</td><td>7,227
(309)</td></tr>
<tr><td>18</td><td>Glc1Man9GN2</td><td>Manα-2Manα-6
|
Manα-6
| |
Manα-2Manα-3 Manβ-4GlcNAcβ-4GlcNAc-DH
|
Glcα-3Manα-2Manα-2Manα-3</td><td>274
(23)</td></tr>
<tr><td>19</td><td>N1</td><td>Galß-4GlcNAcß-2Manα-6 Fucα-6
| |
Manß-4GlcNAcß-4GlcNAc-DH
|
Manα-3</td><td>18
(21)</td></tr>
<tr><td>20</td><td>N2</td><td>Manα-6
|
Manß-4GlcNAcß-4GlcNAc-DH
|
Galß-4GlcNAcß-2Manα-3</td><td>91
(28)</td></tr>
<tr><td>21</td><td>N4</td><td>Galß-4GlcNAcß-2Manα-6
|
Manß-4GlcNAcß-4GlcNAc-DH
|
Manα-3</td><td>93
(5)</td></tr>
<tr><td>22</td><td>N3</td><td>GlcNAcß-2Manα-6 Fucα-6
| |
Galß-4 Manß-4GlcNAcß-4GlcNAc-DH
|
GlcNAcß-2Manα-3</td><td>12
(18)</td></tr>
</table>

<table>
<tr><td>23</td><td>NGA2</td><td>GlcNAcß-2Manα-6
|
Manß-4GlcNAcß-4GlcNAc-DH
|
GlcNAcß-2Manα-3</td><td>13
(11)</td></tr>
<tr><td>24</td><td>NGA2F</td><td>GlcNAcß-2Manα-6 Fucα-6
| |
Manß-4GlcNAcß-4GlcNAc-DH
|
GlcNAcß-2Manα-3</td><td>61
(7)</td></tr>
<tr><td>25</td><td>NGA2B</td><td>GlcNAcß-2Manα-6
|
GlcNAcß-4Manß-4GlcNAcß-4GlcNAc-DH
|
GlcNAcß-2Manα-3</td><td>5
(7)</td></tr>
<tr><td>26</td><td>NGA3B</td><td>GlcNAcß-2Manα-6
|
GlcNAcß-4Manß-4GlcNAcß-4GlcNAc-DH
|
GlcNAcß-4Manα-3
|
GlcNAcß-2</td><td>18
(0)</td></tr>
<tr><td>27</td><td>NGA4</td><td>GlcNAcß-6
|
GlcNAcß-2Manα-6
|
Manß-4GlcNAcß-4GlcNAc-DH
|
GlcNAcß-2Manα-3
|
GlcNAcß-4</td><td>-</td></tr>
<tr><td>28</td><td>NGA5B</td><td>GlcNAcß-2
|
GlcNAcß-4Manα-6
| |
GlcNAcß-6 |
|
GlcNAcß-4Manß-4GlcNAcß-4GlcNAc-DH
|
GlcNAcß-4Manα-3
|
GlcNAcß-2</td><td>57
(20)</td></tr>
<tr><td>29</td><td>GNMan5BGN2</td><td>Manα-6
|
Manα-3Manα-6
|
GlcNAcß-4Manß-4GlcNAcß-4GlcNAc-DH
|
GlcNAcß-2Manα-3</td><td>15
(26)</td></tr>
<tr><td>30</td><td>NA2</td><td>Galß-4GlcNAcß-2Manα-6
|
Manß-4GlcNAcß-4GlcNAc-DH
|
Galß-4GlcNAcß-2Manα-3</td><td>27
(70)</td></tr>
<tr><td>31</td><td>NA2F</td><td>Galß-4GlcNAcß-2Manα-6 Fucα-6
| |
Manß-4GlcNAcß-4GlcNAc-DH
|
Galß-4GlcNAcß-2Manα-3</td><td>-</td></tr>
<tr><td>32</td><td>NA2FB</td><td>Galß-4GlcNAcß-2Manα-6 Fucα-6
| |
GlcNAcß-4Manß-4GlcNAcß-4GlcNAc-DH
|
Galß-4GlcNAcß-2Manα-3</td><td>1
(24)</td></tr>
</table>

33	NA3	Galß-4GlcNAcß-2Manα-6<br>| Manß-4GlcNAcß-4GlcNAc-DH<br>| Galß-4GlcNAcß-4Manα-3<br>| Galß-4GlcNAcß-2	-
34	NA3-Lex	Galß-4GlcNAcß-2Manα-6<br>| Manß-4GlcNAcß-4GlcNAc-DH<br>| Galß-4GlcNAcß-4Manα-3<br>| Galß-4GlcNAcß-2	96<br>(55)
35	NA4	Galß-4GlcNAcß-6<br>| Galß-4GlcNAcß-2Manα-6<br>| Manß-4GlcNAcß-4GlcNAc-DH<br>| Galß-4GlcNAcß-4Manα-3<br>| Galß-4GlcNAcß-2	20<br>(14)
36	A2F(2-3)	NeuAcα-3Galß-4GlcNAcß-2Manα-6 Fucα-6<br>| | Manß-4GlcNAcß-4GlcNAc-DH<br>| NeuAcα-3Galß-4GlcNAcß-2Manα-3	32<br>(12)
37	A2(2-6)	NeuAcα-6Galß-4GlcNAcß-2Manα-6<br>| Manß-4GlcNAcß-4GlcNAc-DH<br>| NeuAcα-6Galß-4GlcNAcß-2Manα-3	22<br>(20)
38	A3	NeuAcα-3Galß-4GlcNAcß-2Manα-6<br>| Manß-4GlcNAcß-4GlcNAc-DH<br>| NeuAcα-3Galß-4GlcNAcß-4Manα-3<br>| NeuAcα-6Galß-4GlcNAcß-2	14<br>(82)

[a] The oligosaccharide probes are all lipid-linked, and are from the collection assembled in the course of research in Glycosciences Laboratory. DH, designates neoglycolipids (NGLs) prepared from reducing oligosaccharides by reductive amination with the amino lipid, 1,2-dihexadecyl-*sn*-glycero-3-phosphoethanolamine (DHPE) (20).

[b] These are means of fluorescence intensities of duplicate spots printed at the high level of probe arrayed (5 fmol/spot); '–', less than 1; The numbers in brackets are the errors (half of the difference of signal intensities of duplicate spots for each glycan probe).

a

Recombinant protein antigen name	Amino acid sequence (amino acids 63-350)
Recombinant Hyr1 N-terminus fragment	METDTLLLWVLLLWVPGSTGGSGHHHHHHG<br>EVEKGASLFIKSDNGPVLALNVALSTLVRP<br>VINNGVISLNSKSSTSFSNFDIGGSSFTNN<br>GEIYLASSGLVKSTAYLYAREWTNNGLIVA<br>YQNQKAAGNIAFGTAYQTITNNGQICLRHQ<br>DFVPATKIKGTGCVTADEDTWIKLGNTILS<br>VEPTHNFYLKDSKSSLIVHAVSSNQTFTVH<br>GFGNGNKLGLTLPLTGNRDHFRFEYYPDTG<br>ILQLRAAALPQYFKIGKGYDSKLFRIVNSR<br>GLKNAVTYDGPVPNNEIPAVCLIPCTNGPS<br>APESESDLNTPTTSSIGT

b

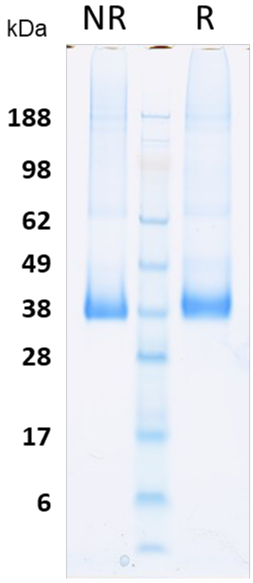

c

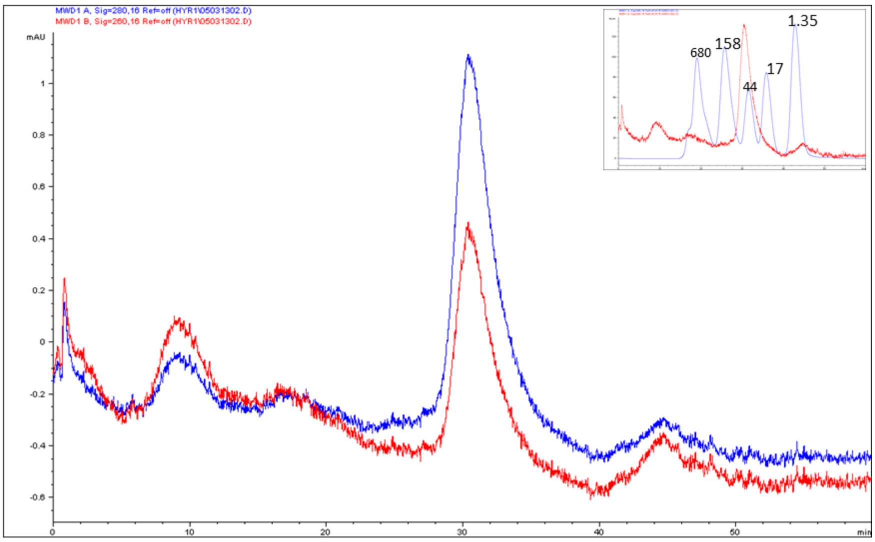

Supplementary Figure 1 – Expression and purification of recombinant Hyr1 protein amino acid sequence. (**a**) Recombinant Hyr1 protein amino acid sequence. Highlighted yellow is the leader sequence, highlighted blue is the 6xHis tag and highlighted red is the linker. Hyr1 protein amino acids 63-350 make up the remainder of the sequence. (**b**) SDS-PAGE gel analysis of purified recombinant Hyr1 protein fragment. (**c**) Chromatograph showing purified recombinant Hyr1 protein fragment following analytical size exclusion chromatography (SEC). NR - non-reduced; R - reduced.

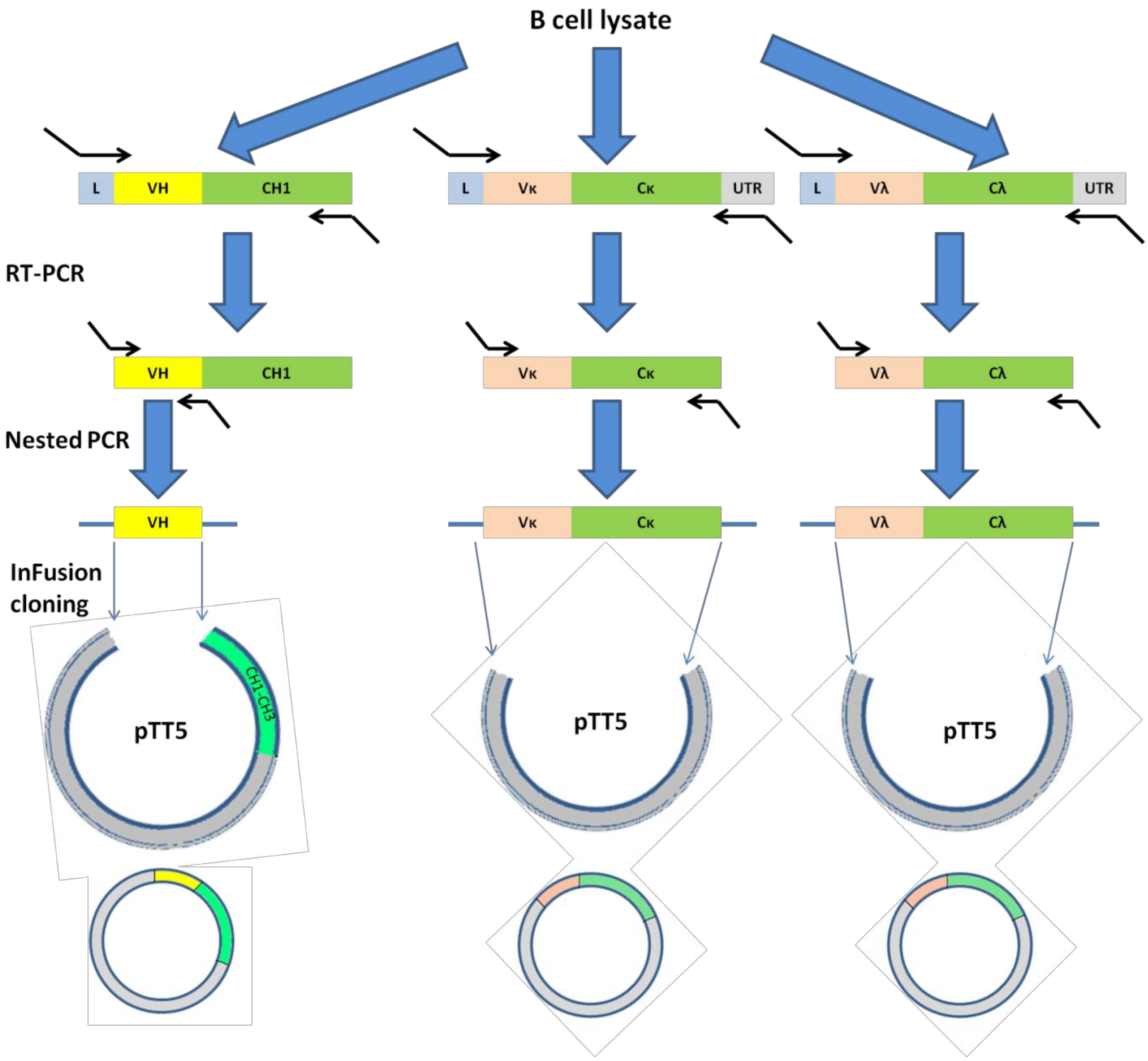

Supplementary Figure 2 – Schematic of VH, Vκ-Cκ and Vλ-Cλ cloning into pTT5 expression vector. B cells positive for antigen binding in the initial ELISA screen were lysed. mRNA in B cell lysate was used as a template for VH, Vκ-Cκ and Vλ-Cλ gene amplification via RT-PCR. RT-PCR was carried out using forward primers specific to human V domain leader sequences and reverse primers specific for human IgCH1, Cκ or Cλ regions or light chain UTR. To increase the specificity of gene amplification, nested PCR was carried out using RT-PCR products as the template. Forward primers specific for human VH FW1 sequences and reverse primers specific for human VH FW4 sequences were used to amplify VH genes. To capture Vκ-Cκ and Vλ-Cλ genes, forward primers specific to human Vκ and human Vλ FW1 sequences were used in combination with reverse primers specific to the 3' end of the human Cκ or human Cλ regions. Primers used in nested PCR reactions contained 15 bp extensions which were complementary to the pTT5 expression vector to facilitate downstream Infusion cloning. Amplification of VH, Vκ-Cκ and Vλ-Cλ genes were done in separate reactions. RT-PCR – reverse transcriptase polymerase chain reaction; UTR untranslated region; L – leader sequence; V_H – heavy chain variable domain; Vκ – kappa chain variable domain; Vλ – lambda chain variable domain; C_H – heavy chain constant domain; Cκ – kappa chain constant domain; Cλ – lambda chain constant domain.

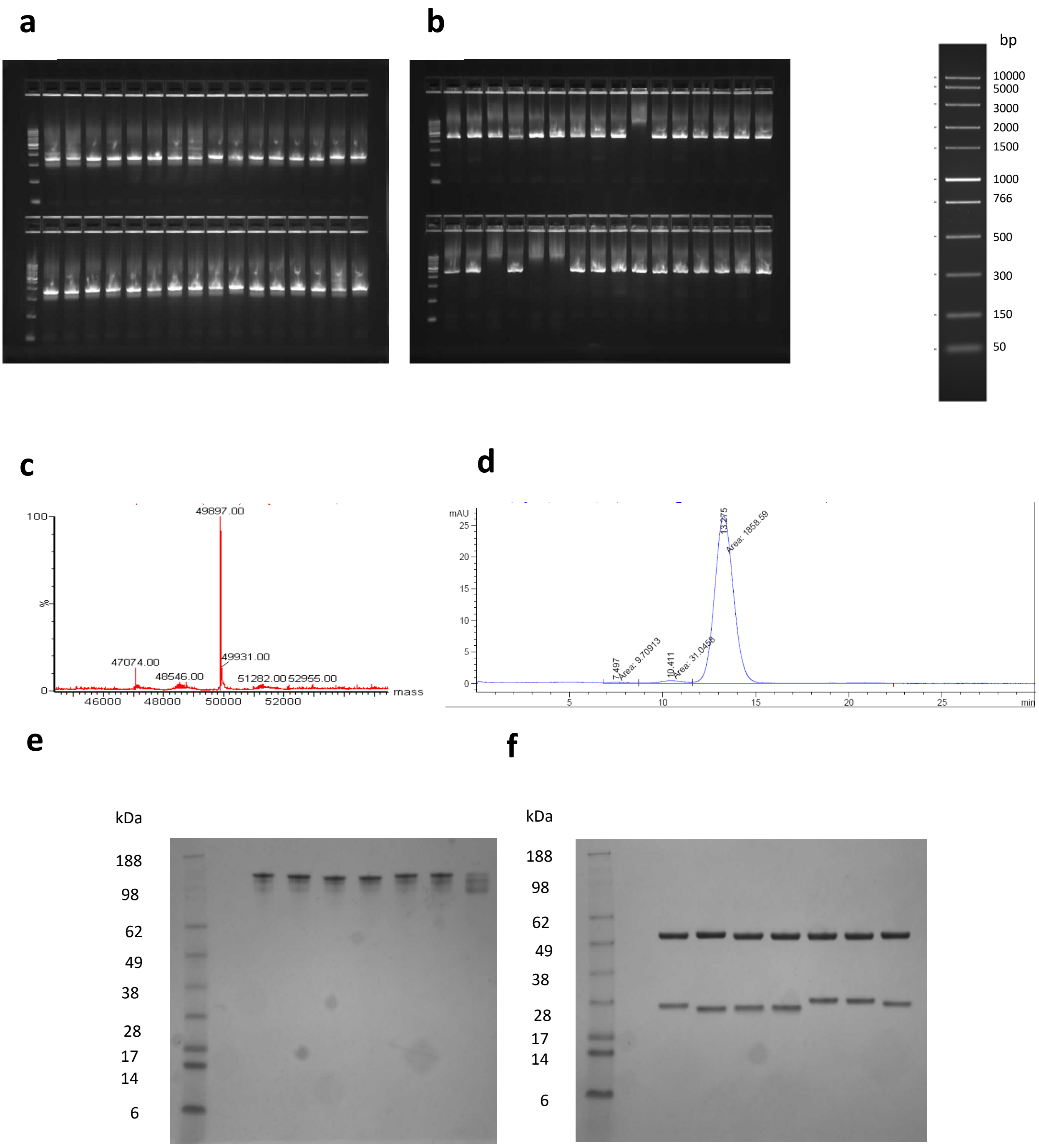

Supplementary Figure 3 - Main stages of the generation of fully human anti-*Candida* mAbs. (**a, b**) Representative agarose gel images following RT-PCR and nested PCR of VH and Vk-Ck genes respectively. (**c, d**) An example of the quality control carried out on the purified recombinant IgG1 mAbs via analytical mass spectrometry of full length de-glycosylated IgG1 (**c**) and analytical SEC (**d**). Further quality control was carried out by SDS-PAGE gel analysis under non-reducing and reducing conditions as shown in (**e**) and **(f**) respectively.

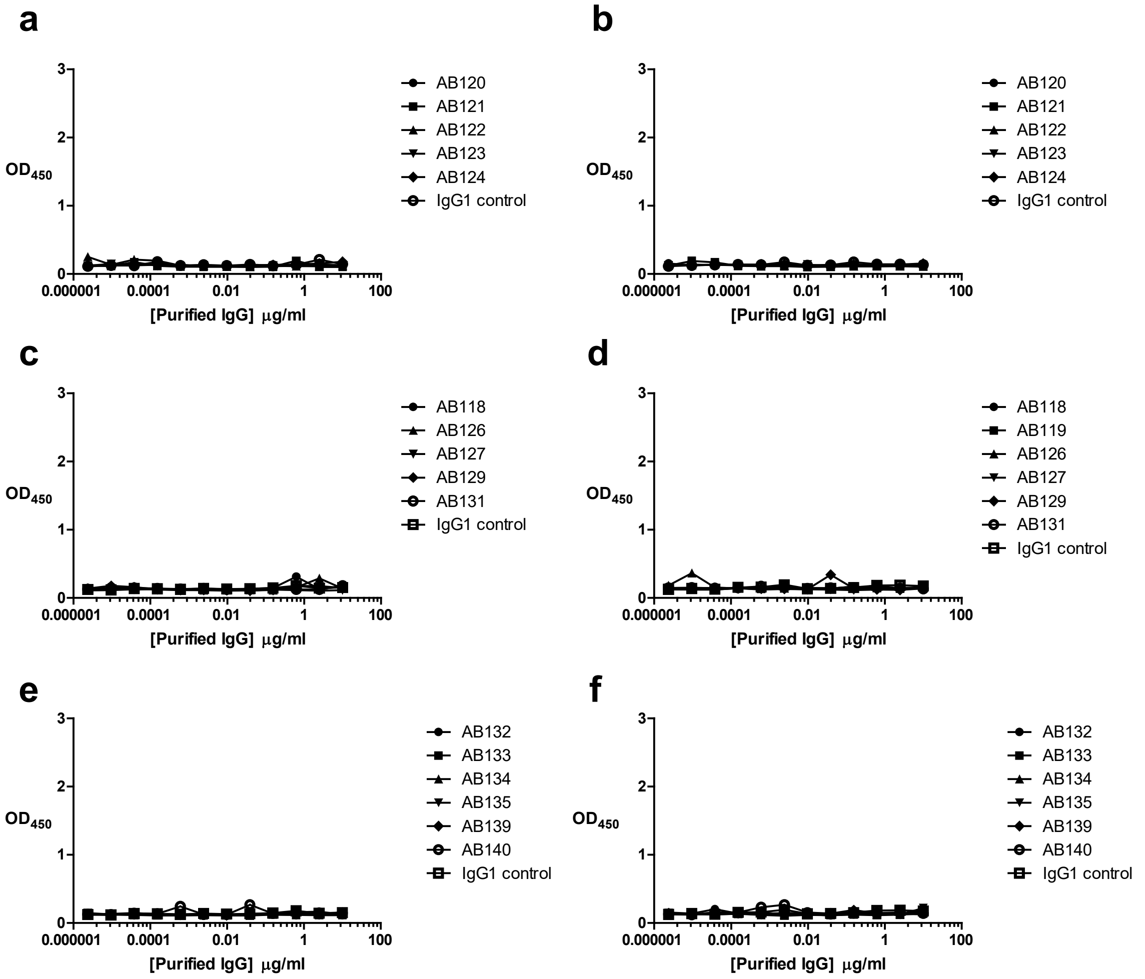

Supplementary Figure 4 - Concentration response curves of purified anti-Hyr1 mAbs screened for binding to unrelated proteins. (**a, b**) Purified anti-Hyr1 mAbs screened against HSA and HEK NA respectively via ELISA. (**c, e**) Purified cell wall mAbs screened against HSA. (**d, f**) Purified cell wall mAbs screened against HEK NA via ELISA. Values represent mean (n=2-4).

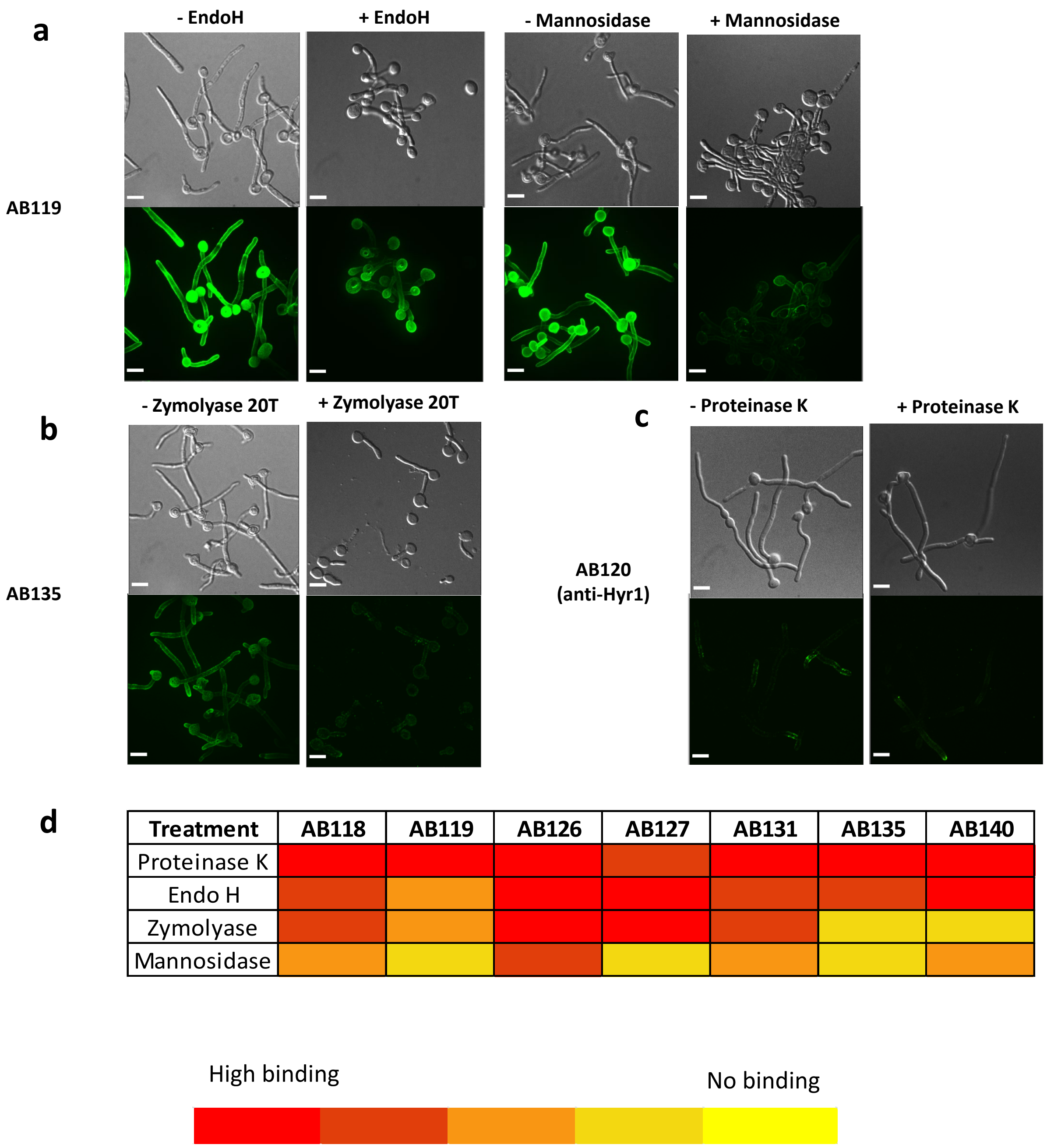

Supplementary Figure 5 – Indirect immunofluorescence of mAbs binding to WT CAI4-Clp10 before and after enzymatic modification of the cell wall. Endoglycosidase H treatment was used to reduce N-linked glycans on the CAI4-Clp10 cell wall; α-mannosidase treatment was used to cleave α-linked mannose from the cell wall; Proteinase K treatment was used to reduce protein residues; Zymolyase 20T enzyme was used to digest ß-1,3-glucans. Shown are example images of AB119 (**a**), AB135 (**b**) and AB120 (**c**) binding to WT *C. albicans* CAI4-Clp10 before/after enzymatic treatment with Endoglycosidase-H, α-mannosidase, Zymolyase 20T and Proteinase K respectively. (**d**) Binding of anti-whole cell mAbs to WT *C. albicans* CAI4-Clp10 cells following the different enzymatic treatments depicted in a heatmap. Decrease in indirect immunofluorescence after enzymatic treatments suggested the nature of the mAb epitopes. A fluorescently conjugated secondary goat anti-human IgG antibody was used to detect anti-*Candida* mAb binding. Scale bars represent 7 µm.

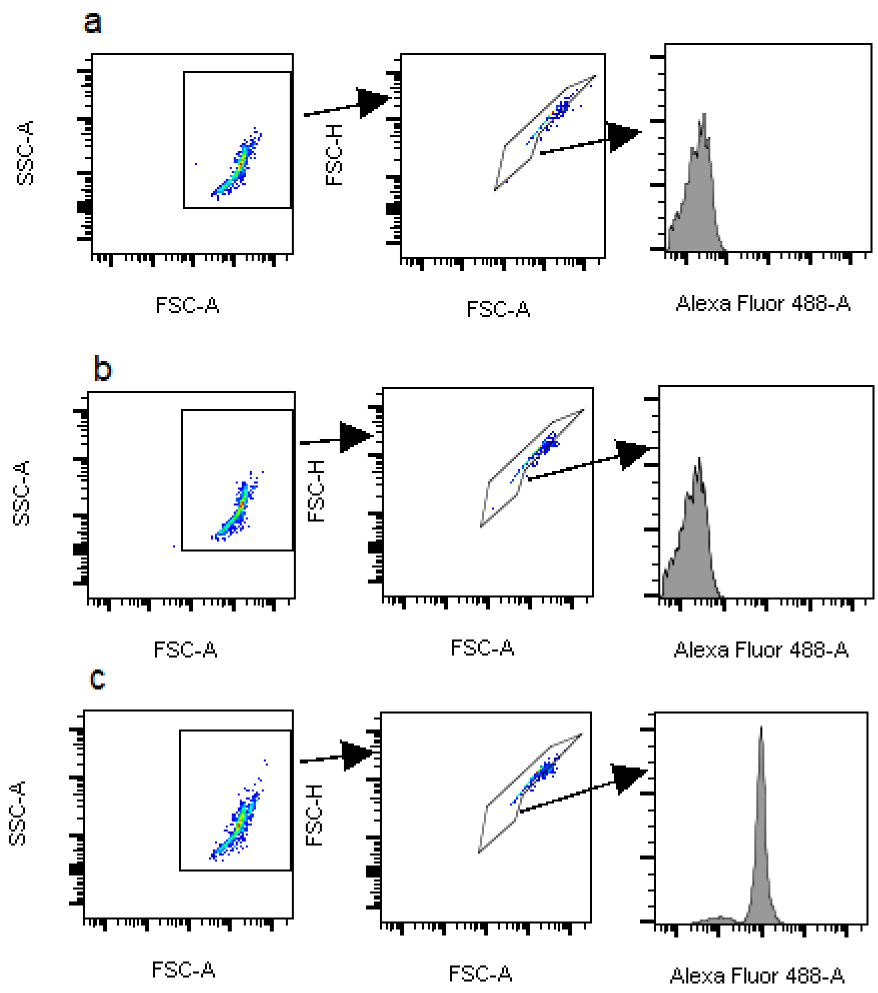

Supplementary Figure 6 – Gating strategy used to determine mAb binding to *Candida* cells via FACS. Panels represent gating strategy employed to identify single cells (middle panels) in antibody positive (AF488^{+}) cell populations (left panels). Median fluorescence intensity (right panels) was determined from the single cell population. (**a**) unstained control sample with *C. albicans*; (**b**) secondary antibody control sample with *C. albicans*; and (**c**) AB135 with *C. albicans*. The same strategy was used to determine mAb binding to all *Candida* and *Saccharomyces* species tested. These data are presented in Figures 5 and 6.

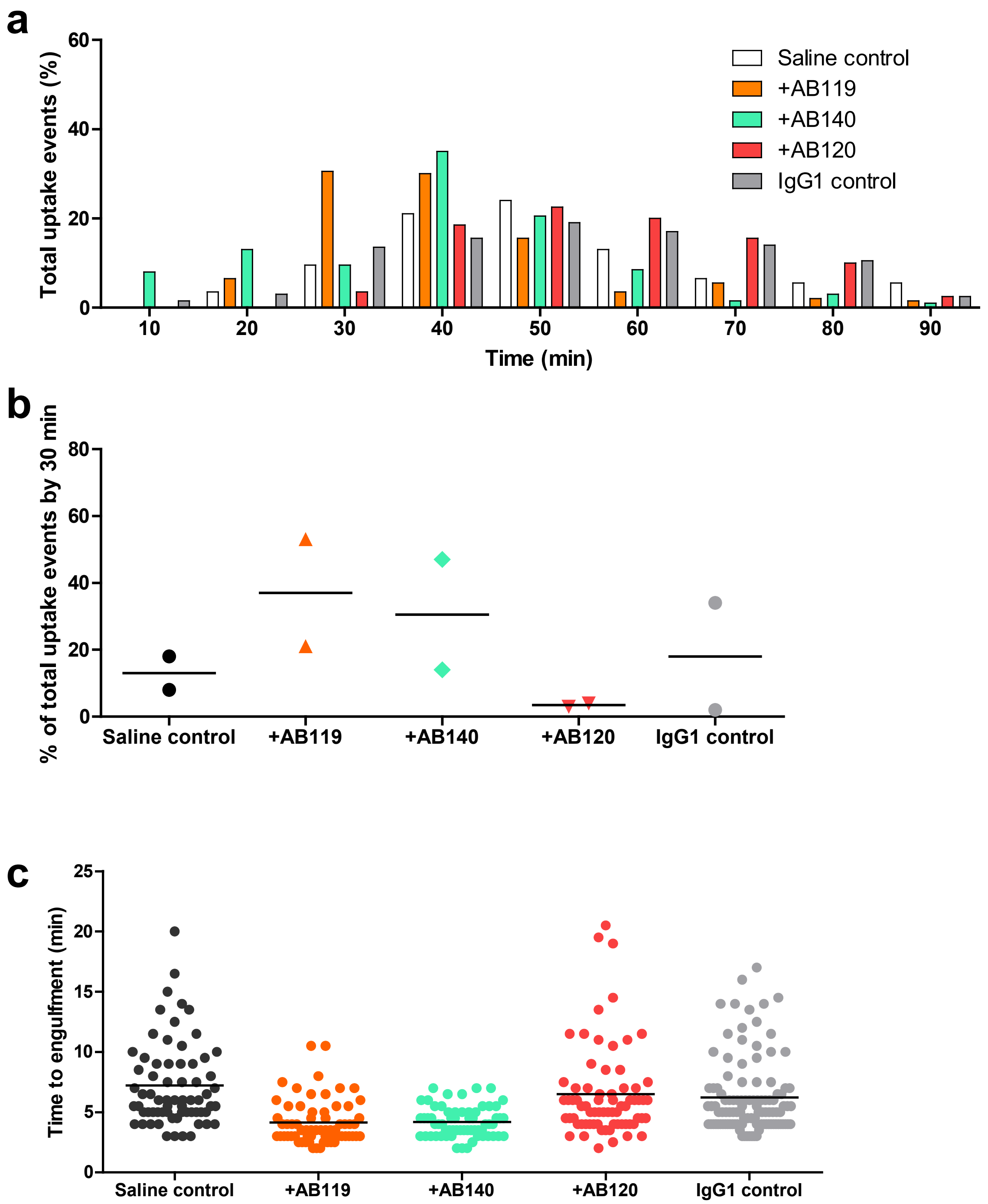

Supplementary Figure 7 – Human monocyte-derived macrophage phagocytosis of live *C. albicans* cells pre-incubated with saline, isotype control mAb or anti-*Candida* mAb. (**a**) Time at which an uptake event occurred over the first 90 min of the assay following *C. albicans* pre-incubation with saline, an IgG1 control antibody, an anti-whole cell reactive mAb (AB119 and AB140) or an anti-Hyr1 mAb (AB120). Bars represent percentage of uptake events (n = 2). (**b**) Percentage of these uptake events that occurred within the first 30 min of the assay. Dots represent average from individual experiments, line represents average (n = 2) and (**c**) average time taken for a macrophage to engulf a live *C. albicans* cell following pre-incubation with saline, an IgG1 control antibody, an anti-whole cell mAb (AB119 and AB140) or an anti-Hyr1 mAb (AB120) at a MOI of 3 (n = 2).

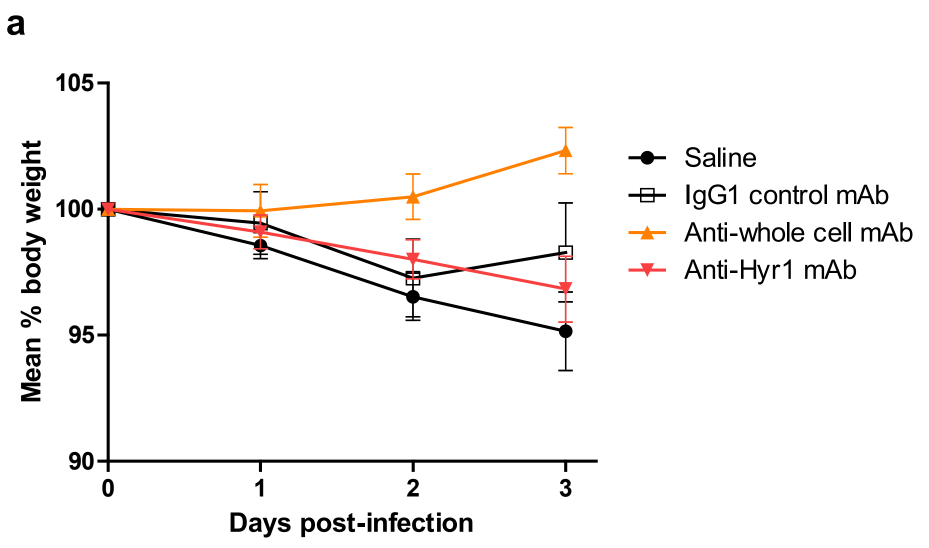

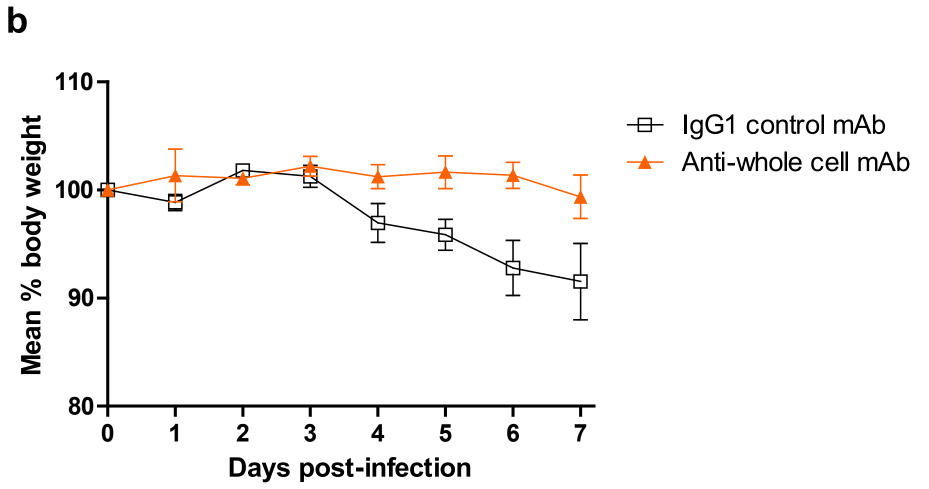

Supplementary Figure 8 – Change in mouse body weight during disseminated candidiasis infection. (**a**) *C. albicans* SC5314 was pre-incubated with saline, IgG1 control, anti-whole cell mAb (AB119) or anti-Hyr1 mAb (AB120) and then injected iv into the tail vein of female BALB/c mice (n=6 per group). (**b**) IgG1 control or anti-whole cell mAb (AB119) was administered ip 4 h prior to injection of *C. albicans* SC5314 iv into the lateral tail vein of male CD1 mice (n=10 per group). Dots represent the mean body weight as a percentage of mouse starting body weight ± SEM.

Supplementary Note: Glycan Microarray Document

Based on (21) MIRAGE Glycan Microarray Guidelines - Beilstein-Institut

Classification	Guidelines
**1. Sample: Glycan Binding Sample**	
Description of Sample	Sample names:<br>Anti-*C. albicans* cell wall mAbs AB118, AB119, AB126, AB127, AB131, AB135, AB140, anti-Hyr1 mAb AB121 are described in the main text.<br><br>Origins: recombinant<br><br>Method of preparation:<br>MAbs AB118-140 were purified recombinant human IgG1 mAbs generated using the single B cell technology. Please see 'Methods' section in the main text for details.
Sample modifications	Not relevant.
Assay protocol	Please see method section in the main text.
**2. Glycan Library**	
Glycan description for defined glycans	The 'Fungal and Bacterial polysaccharide Array' contained 19 saccharides (polysaccharides or glycoproteins) and one lipid-linked neoglycolipid (NGL) probe. The probe names and the predominant oligosaccharide sequences are in **Supplementary Table 3**.<br>The '*N*-glycan Array Set 3' contained 38 sequence-defined *N*-glycan related NGL probes. The probe names and sequences are in **Supplementary Table 4**.<br>The glucan polysaccharides were described previously (Palma A.S, et al. Mol. Cell Proteomics. 2015). The sources of other fungal saccharides (polysaccharides and glycoproteins) are in the 'Methods' section. The antigen preparations from *Mycobacterium smegmatis* and *Mycobacterium tuberculosis* were obtained from the NIH Biodefense and Emerging Infections Research Resources Repository (Beiresources) and were described previously (Hanashima, S. et al. Chembiochem. 2015).<br>The NGL probes are from the collection assembled in the course of research in the Glycosciences Laboratory (https://glycosciences.med.ic.ac.uk/glycanLibraryList.html).
Glycan description for undefined glycans	Not relevant.
Glycan modifications	Polysaccharides and glycoproteins were not modified.

	For NGLs, unless otherwise specified these were prepared from reducing oligosaccharides by reductive amination with the amino lipid, 1,2-dihexadecyl-*sn*-glycero-3-phosphoethanolamine [(DHPE) (Chai et al., Methods Enzymol. 2003)]; AO, NGLs prepared from reducing oligosaccharides by oxime ligation with an aminooxy functionalized DHPE [(AOPE) (Liu et al., Chem. Biol. 2007)].<br>For full description on the definition of lipid moieties of the glycan probes please see https://glycosciences.med.ic.ac.uk/docs/lipids.pdf
**3. Printing Surface; e.g., Microarray Slide**	
Description of surface	Nitrocellulose-coated glass microarray slides.
Manufacturer	'Fungal and Bacterial polysaccharide Array': 16-pad UniSart® 3D slide from Sartorius (Goettingen, Germany)<br>'*N*-glycan Array Set 3': 16-pad Nexterion® Slide from SCHOTT (Jena, Germany)
Custom preparation of surface	Not relevant.
Non-covalent Immobilisation	The lipid-linked oligosaccharide probes were formulated as liposomes by adding carrier lipids, phosphatidylcholine and cholesterol (Liu et al., Methods Mol. Biol. 2012) for arraying and non-covalent immobilisation on nitrocellulose-coated glass slides.<br>Polysaccharides and glycoproteins were immobilised non-covalently without any formulation.
**4. Arrayer (Printer)**	
Description of Arrayer	Nano-Plotter 2.1 (GeSiM, Radeberg, Germany) for the printing of the 'Fungal and Bacterial polysaccharide Array';<br>Piezorray (PerkinElmer LAS, Beaconsfield, UK) for the printing of the '*N*-glycan Array Set 3'.
Dispensing mechanism	Non-contact liquid delivery with four dispensing tips.
Glycan deposition	Approximately 0.33 nl was printed per spot.<br>Polysaccharides and glycoproteins were printed at 0.03 and 0.1 ng per spot, and NGLs at 2 and 5 fmol per spot, all in duplicate.
Printing conditions	The printing solutions for NGLs, polysaccharides and glycoproteins were all aqueous.<br>The NGL printing solutions contained 100 pmol/µl of phosphatidylcholine and cholesterol (both from SIGMA) as lipid carriers in addition to the lipid-linked glycan probes in water (HPLC grade). The concentrations of the NGL probes were 5 and 15 pmol/µl for the 2 and 5 fmol per spot levels, respectively.<br>The printing solutions of polysaccharides/glycoproteins contained polysaccharides or glycoproteins at 0.1 and 0.3 mg/ml for the 0.03

	and 0.1 ng per spot levels, respectively.<br>The printing solutions also contained Cy3 NHS ester (GE Healthcare) at 20 ng/ml (26 fmol/µl) as a marker to monitor the printing process.
**5. Glycan Microarray with "Map"**	
Array layout	The arrayed slides contained 16 identical pads (subarrays). Each pad was set up for printing 64 probes maximum, each at 2 levels in duplicate (four spots for one probe in a row); 256 spots (16x16) in total for 64 probes.<br>The 'Fungal and Bacterial polysaccharide Array' contained 19 probes and the '*N*-glycan Array Set 3' contained 38 NGL probes. The remaining space in each pad was treated as 'blank' when using a grid of 16x16 per pad during the quantitation process; signals from these 'blank probes' were excluded for final data presentation.
Glycan identification and quality control	The 'Fungal, and Bacterial Polysaccharide Array' was analysed with murine Dectin-1 (Palma, et al. Mol. Cell Proteomics. 2015) for quality control purposes in this study. Other data with sequence-specific proteins are available. These include: 1) monoclonal anti-dextran antibodies and carbohydrate-binding modules of bacterial glycoside hydrolases with specificity for α-glucans (TmCBM41) and β-glucans (CmCBM6-2, CtCBM11 and CmCBM32-2); these proteins were used in Palma, et al. Mol. Cell Proteomics. 2015; 2) anti-β1,3-glucan and anti-β1,3/β1,4-glucan antibodies (Biosupplies). Predicted signals were recorded. The biotinylated plant lectin *Aleuria aurantia* lectin (AAL) was also included and gave binding to fucose-containing polysaccharide (Glucurono-XyloMannan).<br>The '*N*-glycan Array Set 3' was analysed with the broadly neutralising anti-HIV mAb PGT128 (Pejchal, et al., Science. 2011) for quality control purposes in this study. Other data include broadly neutralising anti-HIV mAb PGT121 (Falkowska et al., Immunity. 2014) and the biotinylated lectins *Ricinus Communis Agglutinin* I (RCA-120), wheat germ agglutinin (WGA), and Concanavalin A (ConA) (Vector Labs). Predicted binding data were recorded.
**6. Detector and Data Processing**	
Scanning hardware	GenePix 4300A (Molecular Devices, UK)
Scanner settings	Scanning resolution: 10 µm / pixel (this resolution is adequate for the sizes of sample spots)<br>Laser channel: Red (scan wavelength 635 nm)<br>PMT: 350<br>Scan power: 10%
Image analysis software	GenePix® Pro 7 (Molecular Devices)
Data processing	The gpr file was entered into an in-house microarray database using software (designed by Mark Stoll, http://www.beilstein-institut.de/en/publications/proceedings/glyco-2009) for data

	processing. No particular normalisation method or statistical analysis was used.
**7. Glycan Microarray Data Presentation**	
Data presentation	The microarray binding results are in **Fig. 4** and in **Supplementary Tables 3 and 4**.
**8. Interpretation and Conclusion from Microarray Data**	
Data interpretation	Software or algorithms were not used to interpret processed data.
Conclusions	Among the polysaccharides tested, Anti-*Candida* mAbs (except the protein-specific anti-Hyr1 mAb AB121 that did not bind to any of the probes) are shown to bind selectively the *C. albicans N*-mannoprotein.<br>These mAbs showed negligible or no binding to mammalian type *N*-glycan sequences included in the array.

Supplementary References

(1) Brand, A., MacCallum D.M., Brown A.J.P., Gow N.A.R., Odds F.C. Ectopic expression of URA3 can influence the virulence phenotypes and proteome of *Candida albicans* but can be overcome by targeted reintegration of URA3 at the RPS10 locus. *Eukaryot. Cell* **3,** 900-909 (2004).

(2) Bailey, D.A., Feldmann P.J.F., Bovey M., Gow N.A.R., Brown A.J.P. The *Candida albicans HYR1* gene, which is activated in response to hyphal development, belongs to a gene family encoding yeast cell wall proteins. *J. Bacteriol.* **178,** 5353-5360 (1996).

(3) Fonzi, W.A., Irwin M.Y. Isogenic strain construction and gene mapping in *Candida albicans*. *Genetics* **134,** 717-728 (1993).

(4) Gillum, A.M., Tsay E.Y.H., Kirsch D.R. Isolation of the *Candida albicans* gene for orotidine-5'-phosphate decarboxylase by complementation of *S. cerevisiae* ura3 and E. coli pyrF mutations. *Mol. Gen. Genet.* **198,** 179-182 (1984).

(5) Odds, F.C. et al. One year prospective survey of *Candida* bloodstream infections in Scotland. *J. Med. Microbiol.* **56,** 1066-1075 (2007).

(6) Rudek, W. Esterase activity in *Candida* species. *J. Clin. Microbiol.* **8,** 756-759 (1978).

(7) Moran, G.P. et al. Identification and expression of multidrug transporters responsible for fluconazole resistance in *Candida dubliniensis*. *Antimicrob. Agents Chemother.* **42,** 1819-1830 (1998).

(8) Netea, M.G. et al. *Aspergillus fumigatus* evades immune recognition during germination through loss of toll-like receptor-4-mediated signal transduction. *J. Infect. Dis.* **188,** 320-326 (2003).

(9) Satoh, K. et al. *Candida auris* sp. nov., a novel ascomycetous yeast isolated from the external ear canal of an inpatient in a Japanese hospital. *Microbiol. Immunol.* **53,** 41-44 (2009).

(10) Khan, Z.U. et al. Outbreak of fungemia among neonates caused by *Candida haemulonii* resistant to amphotericin B, itraconazole, and fluconazole. *J. Clin. Microbiol.* **45,** 2025-2027 (2007).

(11) Nielsen, K. et al. Sexual cycle of *Cryptococcus neoformans* var. *grubii* and Virulence of congenic a and a isolates. *Infect. Immun.* **71,** 4831-4841 (2003).

(12) Fyfe, M., W. Black and M. Romney. Unprecedented outbreak of *Cryptococcus neoformans* var. *gattii* infections in British Columbia, Canada.Abstracts of the 5th International Conference on Cryptococcus and Cryptococcosis (2002).

(13) Takahara, K. et al. Difference in fine specificity to polysaccharides of *Candida albicans*: Mannoprotein between mouse SIGNR1 and human DC-SIGN. *Infect. Immun.* **80,** 1699-1706 (2012).

(14) Masuoka, J. Surface glycans of *Candida albicans* and other pathogenic fungi: Physiological roles, clinical uses, and experimental challenges. *Clin. Microbiol. Rev.* **17,** 281-310 (2004).

(15) Lowman, D.W. et al. Mannan structural complexity is decreased when *Candida albicans* is cultivated in blood or serum at physiological temperature. *Carbohydr.Res.* **346,** 2752-2759 (2011).

(16) Thornton, C.R. Development of an immunochromatographic lateral-flow device for rapid serodiagnosis of invasive aspergillosis. *Clin. Vaccine Immunol.* **15,** 1095-1105 (2008).

(17) Mishra, A.K., Driessen N.N., Appelmelk B.J., Besra G.S. Lipoarabinomannan and related glycoconjugates: Structure, biogenesis and role in *Mycobacterium tuberculosis* physiology and host-pathogen interaction. *FEMS Microbiol. Rev.* **35,** 1126-1157 (2011).

(18) Dobos, K.M., Khoo K.-., Swiderek K.M., Brennan P.J., Belisle J.T. Definition of the full extent of glycosylation of the 45-kilodalton glycoprotein of *Mycobacterium tuberculosis*. *J. Bacteriol.* **178,** 2498-2506 (1996).

(19) Liu, Y. et al. Neoglycolipid probes prepared via oxime ligation for microarray analysis of oligosaccharide-protein Interactions. *Chem Biol.* **14,** 847-859 (2007).

(20) Chai, W., Stoll M.S., Galustian C., Lawson A.M., Feizi T. Neoglycolipid technology: Deciphering information content of glycome. *Methods Enzymol.* **362,** 160-195 (2003).

(21) Liu, Y. et al. The minimum information required for a glycomics experiment (MIRAGE) project: Improving the standards for reporting glycan microarray-based data. *Glycobiology* **27,** 280-284 (2017).
