# Supplementary material for: Single human B cell-derived monoclonal anti-Candida antibodies enhance phagocytosis and protect against disseminated candidiasis
Source: Nat Commun. 2018 Dec 11;9:5288. doi: 10.1038/s41467-018-07738-1 (PMC6290022; doi:10.1038/s41467-018-07738-1)
Supplement: Supplementary file 3 — Description of Additional Supplementary Files [file 41467_2018_7738_MOESM3_ESM.pdf]

Description of Additional Supplementary Files

File Name: Supplementary Movie 1
Description: Macrophage phagocytosis of live C. albicans cells pre-incubated with AB119. Movie shows J774.1 macrophages (stained magenta) phagocytosing live wild-type C. albicans yeast cells (stained green) which had been pre-incubated with AB119. Movie captures the first 60 min of the interaction. Scale bar = 13 µm.

File Name: Supplementary Movie 2
Description: Macrophage phagocytosis of live C. albicans cells pre-incubated with AB140. Movie shows J774.1 macrophages (stained magenta) phagocytosing live wild-type C. albicans yeast cells (stained green) which had been pre-incubated with AB140. Movie captures the first 60 min of the interaction. Scale bar = 13 µm.

File Name: Supplementary Movie 3
Description: Macrophage phagocytosis of live C. albicans cells pre-incubated with AB120. Movie shows J774.1 macrophages (stained magenta) phagocytosing live wild-type C. albicans yeast cells (stained green) which had been pre-incubated with AB120. Movie captures the first 60 min of the interaction. Scale bar = 14 µm.

File Name: Supplementary Movie 4
Description: Macrophage phagocytosis of live C. albicans cells pre-incubated with control IgG1 mAb. Movie shows J774.1 macrophages (stained magenta) phagocytosing live wildtype C. albicans yeast cells (stained green) which had been pre-incubated with control IgG1 mAb. Movie captures the first 60 min on the interaction. Scale bar = 11 µm.

File Name: Supplementary Movie 5
Description: Macrophage phagocytosis of live C. albicans cells pre-incubated with saline control. Movie shows J774.1 macrophages (stained magenta) phagocytosing live wild-type C. albicans yeast cells (stained green) which had been pre-incubated with saline. Movie captures the first 60 min of the interaction. Scale bar = 17 µm.

File Name: Supplementary Movie 6
Description: Macrophage phagocytosis of live C. albicans filamentous cells pre-incubated with saline control. Movie shows J774.1 macrophages (stained magenta) phagocytosing live wild-type C. albicans filamentous cells (stained green) which had been preincubated with saline. Movie captures the first 60 min of the interaction. Scale bar = 10 µm.

File Name: Supplementary Movie 7
Description: Macrophage phagocytosis of live C. albicans filamentous cells pre-incubated with AB120. Movie shows J774.1 macrophages (stained magenta) phagocytosing live wild-type C. albicans filamentous cells (stained green) which had been pre-incubated with AB120. Movie captures the first 60 min of the interaction. Scale bar = 11 µm.

File Name: Supplementary Movie 8
Description: Macrophage phagocytosis of live C. auris cells pre-incubated with AB119. Movie shows J774.1 macrophages (stained magenta) phagocytosing live wild-type C. auris cells

(stained green) which had been pre-incubated with AB119. Movie captures the first 60 min of the interaction. Scale bar = 17 µm.

File Name: Supplementary Movie 9
Description: Macrophage phagocytosis of live C. auris cells pre-incubated with control IgG1 mAb. Movie shows J774.1 macrophages (stained magenta) phagocytosing live wildtype C. auris cells (stained green) which had been pre-incubated with control IgG1 mAb. Movie captures the first 60 min of the interction. Scale bar = 17 µm.
